# Hysteroscopic morcellation for removal of persistent placental remnants in the uterine cornu: Two case reports

**DOI:** 10.1097/MD.0000000000024097

**Published:** 2021-04-23

**Authors:** Fan Yu, Zhimin Li, Yi Wang, Zhen Yue, Yuanyue Zhong, Liqin Zeng

**Affiliations:** a Department of Gynecology, Guangdong Women and Children Hospital, Guangzhou, Guangdong Province; b Department of Obstetrics and Gynecology, Linzhi People's Hospital, Linzhi, Tibet Autonomous Region, China.

**Keywords:** angular pregnancy, hysteroscopic morcellation, MyoSure, placental remnants

## Abstract

**Rationale:**

Hysteroscopic morcellation is an alternative approach for the removal of placental remnants, given its advantages of safety, efficiency and good reproductive outcomes. This superiority can be even more obvious for removing persistent placental remnants in the lateral angle of the uterine cavity after repeated dilation and curettage (D&C) of an angular pregnancy, which is rarely reported.

**Patient concerns:**

Two patients who were both initially misdiagnosed as having missed intrauterine miscarriages underwent repeated suction-assisted D&C procedures and were found to have persistent placental remnants in the lateral angles of the uterine cavity.

**Diagnoses:**

Ultrasound and hysteroscopy evaluations showed that placental remnants in both cases were in the lateral uterine angles and protruding to the interstitial myometrium around the fallopian tube. We corrected the diagnosis to that of angular pregnancy according to a comprehensive consideration of the ultrasound, hysteroscopy and pathology results.

**Interventions:**

We performed MyoSure hysteroscopic morcellation for both patients and the placental remnants were removed completely without any complication.

**Outcomes:**

The patients were both scheduled for a second-look hysteroscopy 1 to 3 months after surgery, which revealed normal morphology of the uterine cavities and tubal ostia. The patients both achieved normal intrauterine pregnancies several months after surgery.

**Lessons:**

Hysteroscopic morcellation is a good alternative approach for the removal of placental remnants and should be considered in cases in which there might be a high risk of incomplete evacuation or a high risk of uterine perforation, especially in cases of angular pregnancy.

## 1 Introduction

An angular pregnancy is defined as a pregnancy that implants in the lateral angle of the uterine cavity, medial to the uterotubal junction. These account for 2% to 4% of all ectopic pregnancies. Women with an angular pregnancy have a rate of spontaneous or missed miscarriage of 38.5%, and the rate of uterine rupture is 13.6%.^[1]^ This situation can be challenging to diagnose and there are risks of misdiagnosing it as a normal intrauterine pregnancy in some cases of missed miscarriage, especially when no abnormal swelling of the uterine cornu can be found by ultrasonography.

Placental remnants often occur after dilation and curettage (D&C), when the angular pregnancy is misdiagnosed as a normal intrauterine pregnancy. There are many methods for removal of placental remnants, such as “blind” D&C, hysteroscopy with D&C, ultrasound-guided curettage, hysteroscopic loop resection, and hysteroscopic morcellation.^[2]^ Hysteroscopic morcellation and loop resection are more efficient for the selective removal of placental remnants under direct visualization.^[3–6]^ As a novel technique, hysteroscopic morcellation is associated with a shorter operative time and possibly lower odds of incomplete lesion removal than loop resection.^[7,8]^ However, its use in cases of placental remnants in patients with an angular pregnancy has rarely been reported.

Here, we present 2 cases of persistent placental remnants in the uterine cornu after repeated suction-assisted D&C procedures, as angular pregnancies had not been diagnosed initially. We performed hysteroscopic morcellation for the 2 patients using the MyoSure device (Hologic Inc., Marlborough, USA) and the placental remnants were removed completely without any complications. Both patients proceeded to have normal intrauterine pregnancies within a few months.

## 2 Case reports

### 2.1 Case 1

#### 2.1.1 Description

**Figure 1:**
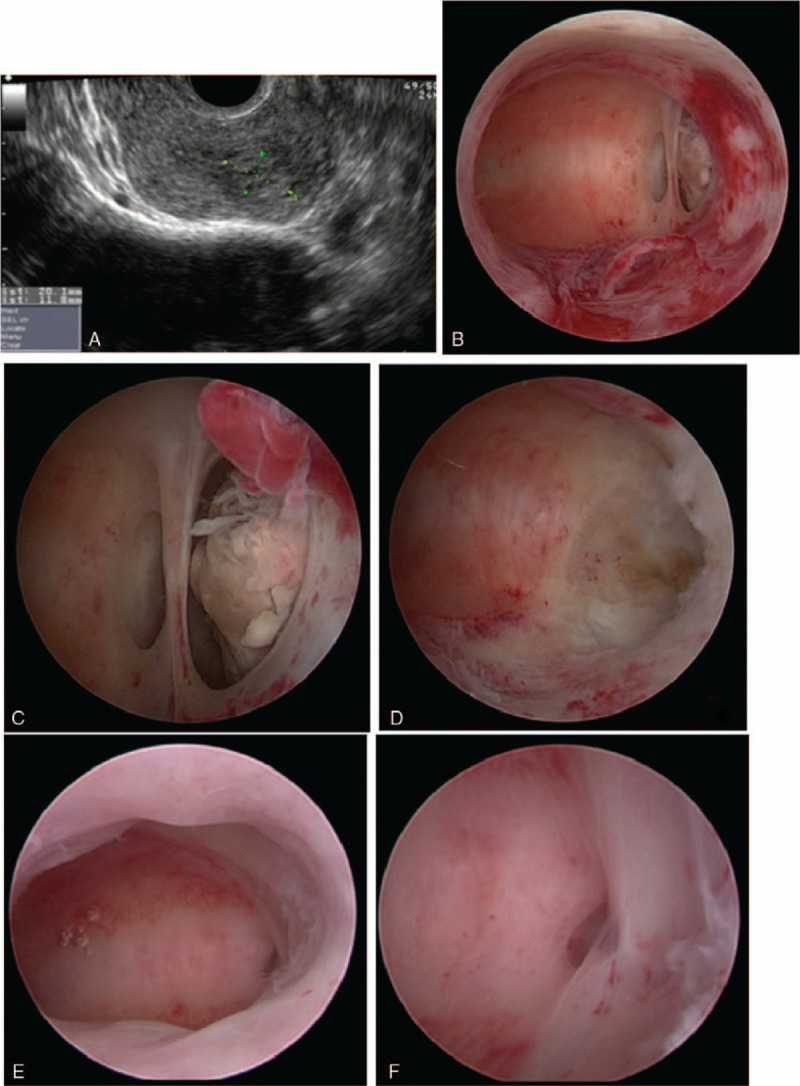
(A) ultrasound image of placental remnants, (B and C) hysteroscopic view of placental remnants in the left uterine horn, (D) hysteroscopic view of left uterine horn after complete resection of placental remnants, (E) second-look hysteroscopic view of uterine cavity, and (F) second-look hysteroscopic view of left uterine horn.

A 28-year-old woman, gravida 3, para 1, was diagnosed with a missed miscarriage at 8 weeks’ gestation in a local hospital. A D&C with vacuum aspiration was performed and the presence of chorionic villi was confirmed by histology. She returned to the same hospital 4 weeks later, complaining of persistent vaginal bleeding. Transvaginal ultrasonography revealed placental remnants and a second suction-assisted D&C was performed together with diagnostic hysteroscopy. This showed the retained placental tissue to be located in the left lateral angle of the uterine cavity with mild adhesions. The evacuation failed because the metal D&C catheter could not reach the retained tissue. Two weeks later, she was referred to our hospital for further treatment. Another transvaginal ultrasound scan was done, which showed a 2.07 ’ 1.18 cm mass of mixed echogenic material suggestive of placental remnants extending to the left angle of the uterus. The left angular myometrial mantle was thinned to approximately 6 mm (Fig. 1A). Her serum beta-human chorionic gonadotropin (β-hCG) test was negative.

#### 2.1.2 Intervention

The hysteroscopic MyoSure morcellation device was introduced and the angular pregnancy was confirmed by a 2 cm area of yellowish-white placental remnants within the left cornual aspect of the uterus. Mild adhesions were found in the left angle (Fig. 1B, C). The retained placental tissue was found intraoperatively to adhere tightly to the uterine wall; nevertheless, it was completely excised stepwise under direct hysteroscopic visualization in 12 minutes (Fig. 1D). The entire procedure is shown in the supplementary video. The total blood loss during surgery was 5 ml with a total normal saline deficit of 100 ml (1800 ml of distension medium was used).

#### 2.1.3 Outcome and follow-up

Histology of the biopsied tissue after surgery confirmed the presence of chorionic villi and decidua tissue. The patient was discharged home the next day without any complications. Three months later, she was scheduled for a second-look hysteroscopy, which revealed a normal uterine cavity with mild membrane adhesion in the left angle (Fig. 1E, F). She was pregnant with a 15-week intrauterine gestation when we started to write this report.

### 2.2 Case 2

#### 2.2.1 Description

**Figure 2:**
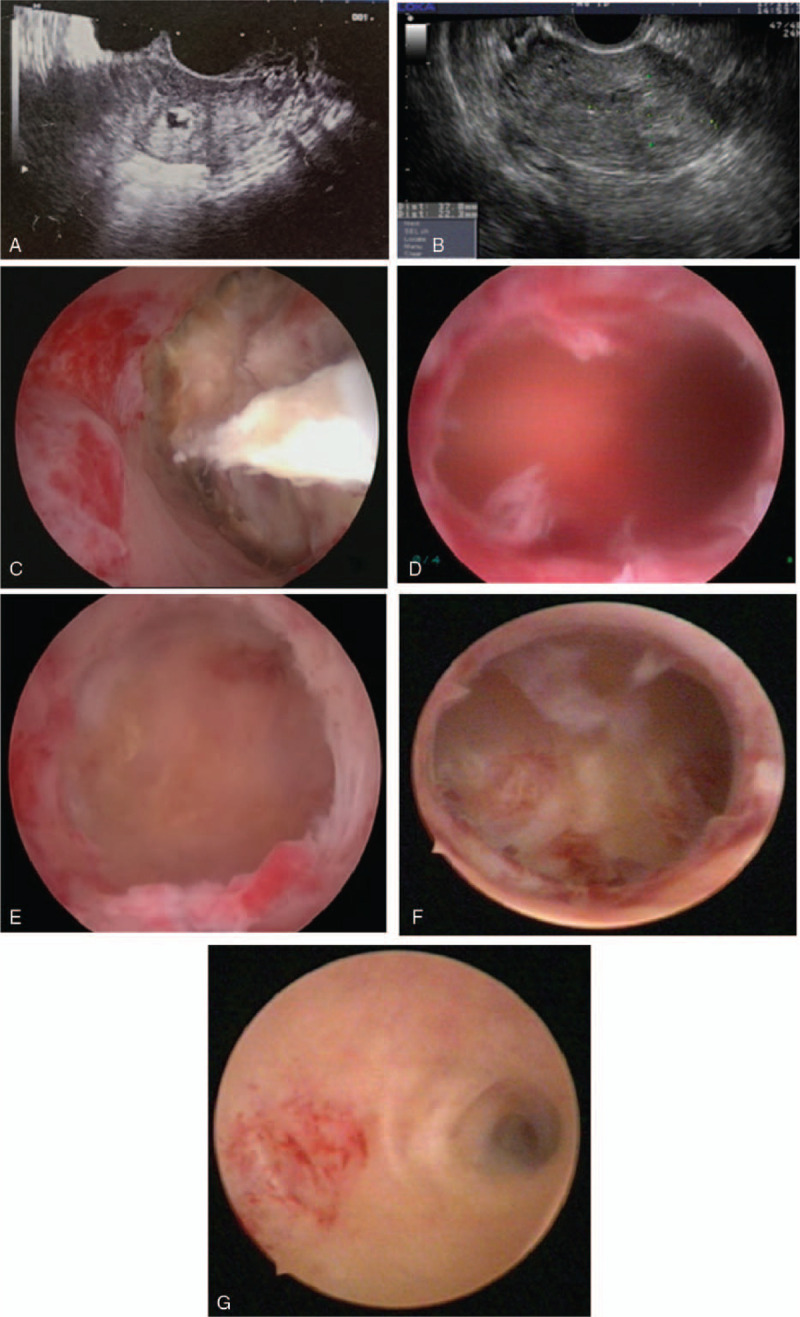
(A) The local ultrasound image of gestational sac, (B) ultrasound image of placental remnants, (C) hysteroscopic view of placental remnants in the left uterine horn, (D) hysteroscopic view of uterine cavity after complete resection of placental remnants, (E) hysteroscopic view of left uterine horn after complete resection of placental remnants, (F) second-look hysteroscopic view of uterine cavity, and (G) second-look hysteroscopic view of left uterine horn.

The second patient was a 30-year-old nulliparous woman with a history of 2 induced abortions and 1 missed miscarriage. This was her fourth pregnancy and it was also diagnosed as a missed miscarriage at 7 weeks’ gestation in a local hospital, as ultrasonography showed an irregular gestational sac with no yolk sac or fetal pole (Fig. 2A). Suction-assisted D&C was performed and a second ultrasound-guided evacuation was performed 8 days later because of placental remnants in the left uterine horn. However, the second evacuation failed as with Case 1 even under ultrasound guidance.

The patient had been bleeding with lower abdominal pain for 4 weeks, so was transferred to our hospital for further management. Transvaginal ultrasonography showed a mixed echogenic shadow measuring 3.78 × 2.23 cm in the uterine cavity expanding to the left horn, which suggested placental remnants. The left angular myometrial mantle was thinned to approximately 4 mm (Fig. 2B). Her serum β-hCG level was 93 IU/l.

#### 2.2.2 Intervention

MyoSure-assisted operative hysteroscopy was performed, which showed a 4 cm area of yellowish-white placental remnants in the left lateral angle of the uterus, protruding to the tubal interstitial myometrium (Fig. 2C). The left uterine horn was significantly enlarged so that it could be mistaken easily for a uterine cavity. The retained placental tissue was found intraoperatively to be tightly adherent to the uterine wall. Old adhesive scars were observed in the fundus of the uterus. The retained tissue was completely removed by hysteroscopic morcellation within a total procedure time of 20 minutes (Fig. 2D, E). The whole procedure is shown in the supplementary video. Total blood loss was 15 ml with a total fluid deficit of 500 ml (3000 ml of distension fluid was used).

#### 2.2.3 Outcome and follow-up

Histology of the biopsied tissues confirmed the presence of chorionic villi and decidua. The patient was discharged home on the first postoperative day without any complications. Cyclic estrogen therapy was suggested for 3 cycles. One month later, the second-look hysteroscopy showed normal morphology of the uterine cavity and tubal ostia and the old adhesive scars in the fundus (Fig. 2F, G). She was pregnant with a 17-week intrauterine gestation when we started to write this report.

## 3 Discussion

Angular pregnancies are not strictly ectopic, as they involve implantation within the uterus. Because of the thin myometrial layer of the uterine cornu and poor trophoblastic development, angular pregnancy may lead to miscarriage or uterine rupture. In some cases, the gestational sacs might expand gradually into the uterine cavity as the pregnancies progress and turn out to be intrauterine. However, in most cases the gestational sacs are more likely to grow towards the myometrium of the uterine cornu.

Diagnosis of an angular pregnancy can be difficult, and it should be distinguished from an intrauterine pregnancy. Ultrasonography is the first-line method for diagnosis before surgery is arranged. Once the pregnancy sac is found to be biased to the uterine horn, the possibility of an angular pregnancy should be considered. The ultrasound image (Fig. 2A) in Case 2 reveals that the gestational sac had expanded to the uterine cornu and the myometrial layer at this location was very thin; however, these features were not noticed by the first doctor. If conventional ultrasonography findings are equivocal, three-dimensional ultrasound or magnetic resonance imaging are important adjuncts to get a better idea of the implant location.^[9]^

The diagnosis of an angular pregnancy also should be distinguished from that of an interstitial pregnancy. The key imaging finding regarding an angular pregnancy is that the gestational sac lies within the endometrium; conversely, an interstitial pregnancy is when the sac lies outside the endometrium with the presence of myometrium noted between the sac and the endometrial cavity.^[1]^ According to the ultrasound images of both our cases, the sacs and placental remnants were intraendometrial, which supported the diagnosis of angular pregnancy. The angular myometrial mantle in Case 2 was thinner than 5 mm, which illustrates that the tissue protruded to the interstitial myometrium. The follow-up hysteroscopies proved that the placental remnants in both cases were in the lateral uterine angles and protruding into the interstitial portion. Thus, ultrasound imaging might require follow-up with hysteroscopy for a final diagnosis especially if angular placental remnants are suspected.

In both our cases, persistent placental remnants were encountered after 2 evacuations using suction-assisted D&C, although the second curettage was performed with diagnostic hysteroscopy (Case 1) or under ultrasound guidance (Case 2). Direct vision-guided removal of uterine pathology is recommended over conventional “blind” D&C to achieve targeted removal of pathological material.^[10]^ Ultrasound-guided evacuation performed in Case 2 would have been helpful for targeting the lesion and detecting the perforation promptly; however this was still not a direct visualization curettage procedure. Additionally, given the concern for perforation of the uterine cornu, this procedure would be performed too conservatively to achieve the targeted tissue. Although diagnostic hysteroscopy with curettage in Case 1 was arranged, the cavity was inspected by hysteroscopy before and after, but not during the procedure, which produced the same result as for Case 2.

Hysteroscopic morcellation and loop resection have been proven to be efficient for the selective removal of placental remnants under direct visualization.^[3–6]^ Both techniques are safe and show high rates of complete removal and tissue availability and low rates of intrauterine adhesions (IUAs).^[3]^ Hysteroscopic morcellation is faster than loop resection because it uses continuous suction of resected chips as it cuts, which keeps the field clear and requires only a single insertion of the instrument.^[3,6]^ Another advantage of the MyoSure instrument is that the outer sheath is only 6.25 mm in diameter and cervical dilation is mostly avoidable. In both cases, the procedure times were short according to the mean procedure time of 11.7 minutes for removing placental remnants of mean diameter of 1.7 cm reported by Hamerlynck et al.^[3]^ In Case 2, the procedure time was 20 minutes; however, the volume of retained placental tissue was large, with a maximum diameter of 3.78 cm.

The angular placental remnants in our 2 cases were noted by hysteroscopy to protrude to the tubal interstitial myometrium. Because of the deep and limited space in the lateral uterine horn and the tightly adhering placental tissues, the evacuation could have been very difficult, especially in Case 1. Nevertheless, the tissues were removed precisely and completely under direct vision with minimal blood loss and without uterine perforation. It is thought that the use of a hysteroscopic morcellator for this indication might reduce the risk of perforation not only by precise removal but also by utilizing the instrument's closed blunted tip and lateral operative window.^[2]^ Furthermore, the MyoSure instrument has a smaller 3-mm outer tubular cutting device, which enabled us to reach tissues in the lateral angle. In our experience, intrauterine pressure should be reduced so that the uterine cavity is just visible. If uterine bleeding obscures the surgical field, oxytocin or pituitrin can be used. In both of our cases, there was minimal bleeding that did not need to be managed further.

As reported in a systematic review,^[5]^ significantly fewer episodes of IUAs were encountered after hysteroscopic evacuation compared with after D&C procedures: 13% vs 30%. The major advantage of hysteroscopic evacuation is the possibility of selective removal of the placental remnants without damaging surrounding healthy endometrium and with a trend towards earlier conception.^[10–12]^ This advantage is more obvious for hysteroscopic morcellation using mechanical excision, which avoids electrical and thermal injury to the endometrium. In both cases, second-look hysteroscopies showed that there were only mild IUAs and old adhesive scars. Moreover, normal intrauterine pregnancies were obtained several months after surgery in both of our cases.

## 4 Conclusion

Our cases demonstrate the value of hysteroscopic morcellation, both in confirming the diagnosis of an angular pregnancy and in completely removing placental remnants from the uterine cornu. Thanks to the advantages of safety, efficiency and good reproductive outcomes, hysteroscopic morcellation has emerged as an alternative approach for the removal of placental remnants, and this method should be considered in cases in which there might be high risks of incomplete evacuation or of uterine perforation, especially in cases of angular pregnancy.

## Acknowledgments

Informed and signed consents were obtained from the patients to publish this case report, which is greatly appreciated.

## Author contributions

**Resources:** Zhimin Li, Yi Wang, Zhen Yue, Yuanyue Zhong, Liqin Zeng.

**Writing – original draft:** Fan Yu.

**Writing – review & editing:** Liqin Zeng.

## Supplementary Material

Supplemental Digital Content

## Supplementary Material

Supplemental Digital Content
